# COVID-19: self-reported reductions in physical activity and increases in sedentary behaviour during the first national lockdown in the United Kingdom

**DOI:** 10.1007/s11332-022-01012-0

**Published:** 2022-10-26

**Authors:** Patrick Swain, Emily James, Jonathan M. Laws, Clare Strongman, Stuart Haw, Gill Barry, Henry C. Chung, Dan Gordon

**Affiliations:** 1 Faculty of Health and Life Sciences, Northumbria University, Newcastle-upon-Tyne, UK; 2 Cambridge Centre for Sport and Exercise Sciences, Anglia Ruskin University, Cambridge, UK

**Keywords:** Physical activity, Sedentary behaviour, Public health, COVID-19

## Abstract

**Purpose:**

The United Kingdom (UK) government imposed its first national lockdown in response to COVID-19 on the 23rd of March 2020. Physical activity and sedentary behaviour levels are likely to have changed during this period.

**Methods:**

An online survey was completed by *n* = 266 adults living within the UK. Differences in day-to-day and recreational physical activity (at moderate and vigorous intensities), travel via foot/cycle, and sedentary behaviour were compared before and during the initial COVID-19 lockdown.

**Results:**

The median level of total weekly physical activity significantly reduced (− 15%, *p* < 0.001) and daily sedentary time significantly increased (+ 33%, *p* < 0.001). The former was caused by a significant reduction in weekly day-to-day physical activity at moderate intensities (*p* < 0.001), recreational activities at vigorous (*p* = 0.016) and moderate (*p* = 0.030) intensities, and travel by foot/cycle (*p* = 0.031). Sub-group analyses revealed that some populations became disproportionally more physically inactive and/or sedentary than others, such as those that were: living in a city (versus village), single (versus a relationship), an athlete (versus non-athlete), or earning an average household income < £25,000 (versus > £25,000).

**Conclusions:**

Now that the UK is transitioning to a state of normal living, strategies that can help individuals gradually return to physical activities, in accordance with the 2020 WHO guidelines, are of paramount importance to reducing risks to health associated with physical inactivity and sedentary behaviour.

## Introduction

The World Health Organization (WHO) declared the novel coronavirus (COVID-19) as a Global Health Emergency [1]. Global government strategies to control the spread of COVID-19 included limiting social interaction using enforced national lockdowns. In the United Kingdom (UK) on March 23rd 2020, the government enforced a national lockdown during which people could leave their household for essential reasons only, such as the collection of food, medicine, and outdoor exercise once per day [2]. The lockdown has a clear rationale behind reducing exposure to and transmission of COVID-19 [3], however, this may have also substantially disrupted individuals' daily routines, including physical activity and sedentary behaviour levels, which are known to impact long-term health [4–6].

The recently updated *World Health Organization 2020 Guidelines on Physical Activity and Sedentary Behaviour* [7] recommends that adults should partake in 150–300 min of moderate-intensity or 75–150 min of vigorous-intensity physical activity (or some combination of both) whilst reducing sedentary behaviour. There is an overwhelming evidence-base in support of physical activity for improving health-related outcomes such as for reducing the risk of all-cause mortality [8] and major non-communicable diseases including several cancers [9], cardiovascular disease [10], type 2 diabetes [11], dementia, and Alzheimer’s [12]. In addition, physical activity is recommended for reducing the impact of mental health disorders such as anxiety and depression [13–15]. Evidence also suggests that reducing levels of sedentary behaviour can bring independent health benefits [16]. Importantly, adults meeting physical activity guidelines have been shown to be at a decreased risk of COVID-19 infection, severe illness, and related death [17]. Despite the benefits of a physically active lifestyle, the opportunity to be active during the UK’s first COVID-19 lockdown was severely limited. Leisure centres and gyms, among other recreational activity facilities were required to close throughout the lockdown, and individuals were limited to exercise outdoors once a day (alone or with members of their household) [2]. Furthermore, the requirement to work from home where possible is likely to increase sedentary behaviours such as sitting, bed rest, and lounging. Public Health England [18] and the WHO [19] encouraged people to remain physically active during the pandemic, however, it is unclear whether this occurred during the first UK lockdown in response to COVID-19.

The present study, therefore, aimed to quantify changes in day-to-day living and recreational physical activity, travel via foot/cycle, and sedentary behaviour levels before and during the first UK lockdown (enforced on 23rd March 2020). It was hypothesized that during lockdown compared to before, total physical activity levels would significantly reduce, and sedentary behaviour levels would significantly increase.

## Materials and methods

### Participants

Four preliminary questions via an online survey confirmed that participants met the following eligibility criteria: (1) ≥ 18 years of age, (2) current resident of the UK, (3) following the government lockdown rules, and (4) no recent changes in health status that could influence physical activity levels. All participants must have provided informed consent was provided by all participants via the online questionnaire. The survey was completed by 266 participants that met all eligibility criteria. Ethical approval was granted by the Anglia Ruskin University Ethics Panel (approval code: SES_STAFF_19-10).

### Online survey

Online Surveys (https://www.onlinesurveys.ac.uk/) was used for the creation and dissemination of the questionnaire. Participant demographics (e.g., sex, age, living location) were initially collected. Thereafter, physical activity levels (frequency and duration) during day-to-day living (e.g., work or household-related labor), recreational exercise, and travel via foot/cycle alongside daily sedentary behaviour duration were collected using a modified version of the WHO Global Physical Activity Questionnaire (GPAQ) Version 2 [20]. Physical activity questions were asked in relation to two intensity domains as defined by the WHO GPAQ: (1) moderate (moderate physical effort that causes small increases in breathing and heart rate for ≥ 10-min), and (2) vigorous (hard physical effort that causes a large increase in breathing or heart rate for ≥ 10-min). The GPAQ has been demonstrated to have fair-to-moderate validity when self-administered and moderate reliability compared to an interviewer-administered version [21].

To allow pre-lockdown versus during-lockdown analysis, physical activity and sedentary behaviour questions were asked twice in the following order: (1) participants were asked to recall their general activity levels in the 8-week prior to the lockdown, and (2) participants were asked to recall their current general activity levels during the lockdown. To reduce the impact of recall bias, especially for numerical data (e.g., duration of sedentary behaviour), the survey was disseminated as soon as possible following the UK lockdown; publicly available from the 10th of April and closed on the 26th of April 2020 (lockdown: 23rd of March 2020). Within the first few days of survey dissemination (10–13th April), 75% of responses were submitted, and by the 17th of April 94% of responses were submitted. To improve readability, information regarding the types of activity in each exercise domain/context was provided alongside the format in which the participant should respond. The survey was initially disseminated using social media platforms (e.g., Facebook) and emails followed by snowball sampling (e.g., participants sharing the survey link with others that expressed interest).

### Data processing

Data were exported to the Statistical Package for Social Sciences (SPSS, Version 26, Chicago, IL). Outliers were removed from physical activity and sedentary behaviour metrics; defined as any data point that was  > 300% of the upper or lower interquartile range for a given measurement at the cohort level (1.3% [*n* = 41] of data points were classified outliers). For all physical activity data, participants had to initially confirm that they did/did not exercise in a particular context (e.g., “did you do any vigorous-intensity sport, fitness or recreational (leisure) activities…” [Yes/No]). If participants selected ‘No’, a null result (valueless character) was generated for the succeeding questions relating to exercise frequency and duration for that activity due to the questionnaire routing system used. To ensure that these data were included in the analysis, they were manually inputted (i.e., frequency = 0 and duration = 0 h). This avoided only including participants that had completed some form of activity, which would have overestimated the cohort’s physical activity levels.

Physical activity frequency per week and duration per session outcomes were multiplied to calculate weekly activity volume (activity volume = frequency [per week] × duration [h]). Additionally, metabolic equivalents (MET-h/week) were calculated from moderate- and vigorous-intensity activity data in accordance with the WHO GPAQ guidelines to estimate participant’s MET-h/week [20]. One MET is defined as the average energy expenditure of an adult sitting quietly; 3.5mlO_2_/kg/min (or 1 kcal/kg/h) [22]. The *2008 Physical Activity Guidelines for Americans* define physical activities at moderate intensities as 3.0–5.9 METs and vigorous intensities as ≥ 6 METs [23]. The WHO GPAQ data processing guidelines are similar, however, are limited to using fixed MET values as opposed to a range, therefore, in accordance with the guidelines, moderate-intensity activity (including travel via foot/cycle) and vigorous-intensity activity are defined as 4 METs and 8 METs, respectively [20]. Therefore, for example, recreational physical activity MET-h/week = (moderate intensity activity volume × 4) + (vigorous-intensity activity volume × 8). MET-h/week was calculated for day-to-day physical activity, travel via foot/cycle, and recreational physical activity independently. The sum of all activity METs was used to estimate total MET-h/week as an indicator of total weekly physical activity levels.

### Statistical analyses

All statistical analyses were conducted using SPSS. All outcomes were assessed for normality via the Shapiro–Wilk Test; none were found to be normally distributed. Physical activity and sedentary behaviour levels for the entire cohort before and during the lockdown were compared using the Wilcoxon Signed Rank Test. Total physical activity levels (MET-hours/week) and sedentary behaviour duration before and during the lockdown were sub-analysed in the following categories: biological sex (male; female), lockdown living location (city; town; village), marital status (single; partnership/married), educational status (degree qualification or above; no degree), lockdown average household annual income (above £25,000; below £25,000) and athletic status (athlete; non-athlete). Comparisons for the absolute change in total physical activity and sedentary behaviour levels between sub-groups were made using the Mann–Whitney *U* Test (2 groups) or Kruskal–Wallis Test (≥ 3 groups). For all analyses, effect sizes (ES) were calculated using Hedge’s G (0.2 = small, 0.5 = medium, 0.8 = large, and 1.3 = very large) [24]. Statistical significance was set at *p* < 0.05. All data are reported as medians and interquartile ranges.

## Results

Table 1 presents participant characteristics. Table 2 presents physical activity levels for the cohort. Table 3 presents the change in total physical activity levels (METs/week) within and between sub-groups. Table 4 presents the change in sedentary behaviour duration for the cohort and also within and between sub-groups.

**Table 1:** Participant characteristics

Characteristic	Frequency (n)	Percentage of sample
*Age (years)*		
18–24	31	11.7
25–34	57	21.4
34–44	40	15.0
45–54	74	27.8
55–64	45	16.9
≥ 65	19	7.1
*Sex*		
Male	68	25.7
Female	197	74.3
*Marital status*		
Single and never married	88	35.6
Civil/domestic partnership or married	159	64.4
*Athletic status*		
Non-athlete	167	63.3
Amateur athlete	97	36.7
*Living location*		
City	65	25.4
Town	91	35.5
Village	100	39.1
*UK area*		
England	256	96.2
Scotland	4	1.5
Wales	5	1.9
Northern Ireland	1	0.4
*Educational status (highest qualification)*		
Degree or above	192	72.2
Below degree	74	27.8
*Average household income*		
Below £25,000	53	20.1
Above £25,000	211	79.9

**Table 2:** Physical activity levels before and during lockdown

Parameter	Before Lockdown	During Lockdown	*n*	*P*	ES
*Vigorous intensity activity during day-to-day living*					
Frequency (days per week)	0.00 (2.00)	0.00 (1.00)	260	0.930	< 0.01
Duration (hours per session)	0.00 (0.25)	0.00 (0.17)	259	0.290	0.13
Volume (hours per week)	0.00 (0.50)	0.00 (0.33)	259	0.444	0.11
METs (per week)	0.00 (4.00)	0.00 (2.64)	259	0.455	0.11
*Moderate intensity activity during day-to-day living*					
Frequency (days per week)	4.00 (5.25)	2.00 (5.00)	250	0.001	0.23^S^
Duration (hours per session)	0.67 (1.50)	0.33 (1.00)	248	< 0.001	0.41^S^
Volume (hours per week)	2.50 (7.00)	1.00 (4.69)	248	< 0.001	0.34^S^
METs (per week)	10.00 (28.00)	4.00 (18.76)	248	< 0.001	0.34^S^
*Travel by foot/cycle*					
Frequency (days per week)	3.00 (6.00)	1.00 (5.00)	247	0.020	0.18
Duration (hours per session)	0.38 (1.00)	0.21 (0.90)	244	0.065	0.15
Volume (hours per week)	1.65 (4.69)	0.50 (3.50)	244	0.032	0.17
METs (per week)	6.67 (18.67)	2.00 (14.00)	244	0.031	0.17
*Vigorous intensity recreational activity*					
Frequency (days per week)	2.00 (4.00)	2.00 (5.00)	240	0.849	0.01
Duration (hours per session)	0.75 (1.00)	0.33 (1.00)	240	< 0.001	0.37^S^
Volume (hours per week)	1.50 (4.00)	1.00 (3.50)	240	0.016	0.16
METs (per week)	12.00 (32.00)	8.00 (28.00)	240	0.016	0.16
*Moderate intensity recreational activity*					
Frequency (days per week)	1.00 (2.00)	0.00 (4.00)	242	0.010	0.18
Duration (hours per session)	0.17 (1.00)	0.00 (0.75)	242	0.123	0.09
Volume (hours per week)	0.21 (2.00)	0.00 (3.00)	242	0.030	0.17
METs (per week)	0.84 (8.00)	0.00 (12.00)	242	0.030	0.17
*Total weekly physical activity*					
Total MET-hours/week	52.00 (69.20)	44.00 (56.30)	207	< 0.001	0.28^S^

Data are presented as median (interquartile range). Data were analysed using the Wilcoxon Signed Rank Test. Effect size thresholds are defined as: 0.2 (small [^S^]), 0.5 (medium [^M^]), 0.8 (large [^L^]), and 1.3 (very large [^VL^])

*MET* metabolic equivalents

**Table 3:** Total physical activity levels before and during lockdown

Group	Before (MET-hrs/wk)	During (MET-hrs/wk)	P	ES	P	ES
*Biological sex*						
Male (*n* = 49)	70.00 (84.28)	46.60 (63.24)	0.022	0.39^S^	0.571	0.20^S^
Female (*n* = 157)	48.00 (60.68)	42.00 (49.28)	0.003	0.25^S^		
*Living area*						
City (*n* = 57)	60.00 (75.74)	42.00 (49.34)	< 0.001	0.61^ M^	0.056	0.53^ M^
Town (*n* = 64)	48.00 (61.42)	41.00 (51.60)	0.143	0.23^S^		0.35^S^
Village (*n* = 81)	52.00 (72.42)	48.00 (72.58)	0.220	0.10		0.15
*Athletic status*						
Non-athlete (*n* = 129)	38.00 (48.33)	36.00 (44.02)	0.082	0.14^S^	0.045	0.27^S^
Athlete (*n* = 76)	79.42 (84.00)	62.30 (81.66)	0.001	0.40^S^		
*Marital status*						
Single (*n* = 70)	64.50 (86.21)	40.30 (55.93)	< 0.001	0.52^ M^	0.003	0.43^S^
Partnered (*n* = 125)	48.00 (66.20)	46.00 (62.02)	0.149	0.15		
*Education status*						
≥ Degree (*n* = 147)	52.00 (63.20)	47.96 (64.00)	0.011	0.23^S^	0.119	0.22^S^
< Degree (*n* = 60)	53.04 (81.75)	40.00 (54.80)	0.004	0.41^S^		
*Avg household income*						
≥ £25,000 (*n* = 167)	50.36 (57.84)	46.48 (56.21)	0.024	0.18	0.009	0.50^ M^
< £25,000 (*n* = 43)	65.00 (96.00)	36.00 (52.60)	< 0.001	0.65^ M^		

Data are presented as median (interquartile range). Data were analysed before and during the lockdown within groups using the Wilcoxon Signed Rank Test. The change in physical activity levels was compared between groups using the Mann–Whitney *U* Test (2 groups) or the Kruskal–Wallis Test (≥ 3 groups). Living area effect sizes (ES) were calculated based on the following comparisons: City vs. Village (ES = 0.53), City vs. Town (ES = 0.35), and Village vs. Town (ES = 0.15). Effect size thresholds are defined as: 0.2 (small [^S^]), 0.5 (medium [^M^]), 0.8 (large [^L^]), and 1.3 (very large [^VL^]). Sub-category ‘Athlete’ only includes those that were amateur and not professional due to the limited sample size of the latter group (*n* = 2)

*MET* metabolic equivalents

**Table 4:** Sedentary behaviour levels before and during lockdown

Group	Before (h/day)	During (h/day)	P	ES	P	ES
All (*n* = 255)	6.00 (5.00)	8.00 (5.50)	< 0.001	0.36^S^	NA	NA
*Biological sex*						
Male (*n* = 65)	7.00 (5.29)	8.00 (7.00)	0.010	0.24^S^	0.234	0.15
Female (*n* = 189)	6.00 (5.00)	7.00 (5.75)	< 0.001	0.42^S^		
*Living area*						
City (*n* = 68)	6.00 (4.75)	9.00 (6.88)	< 0.001	0.42^S^	0.499	0.04
Town (*n* = 84)	6.00 (4.11)	7.50 (5.00)	< 0.001	0.32^S^		0.15
Village (*n* = 95)	5.00 (5.00)	7.00 (6.00)	< 0.001	0.40^S^		0.12
*Athletic status*						
Non-athlete (*n* = 161)	6.00 (4.25)	8.00 (5.00)	< 0.001	0.39^S^	0.639	0.04
Athlete (*n* = 92)	6.00 (5.11)	7.75 (6.00)	< 0.001	0.34^S^		
Marital status						
Single (*n* = 86)	7.00 (4.69)	9.00 (5.06)	< 0.001	0.57^ M^	0.002	0.37^S^
Partnered (*n* = 152)	6.00 (5.00)	6.00 (6.00)	< 0.001	0.26^S^		
*Education status*						
≥ Degree (*n* = 183)	6.00 (5.00)	8.00 (6.00)	< 0.001	0.32^S^	0.079	0.12
< Degree (*n* = 72)	5.00 (4.00)	6.00 (6.38)	< 0.001	0.49^S^		
*Avg household income*						
≥ £25,000 (*n* = 200)	6.00 (5.00)	7.00 (6.00)	< 0.001	0.28^S^	0.002	0.48^S^
< £25,000 (*n* = 54)	6.00 (4.00)	10.00 (6.00)	< 0.001	0.65^ M^		

Data are presented as median (interquartile range). Data were analysed before and during the lockdown within groups using the Wilcoxon Signed Rank Test. The change in physical activity levels were compared between groups using the Mann–Whitney *U* Test (2 groups) or the Kruskal–Wallis Test (≥ 3 groups). Living area effect sizes (ES) were calculated based on the following comparisons: City vs. Village (ES = 0.04), City vs. Town (ES = 0.15), and Village vs. Town (ES = 0.12). Effect size thresholds are defined as: 0.2 (small [^S^]), 0.5 (medium [^M^]), 0.8 (large [^L^]), and 1.3 (very large [^VL^]). Sub-category ‘Athlete’ only includes those that were amateur and not professional due to the limited sample size of the latter group (*n* = 2)

## Discussion

This study aimed to quantify changes in physical activity and sedentary behaviour levels before and during the first UK COVID-19 lockdown imposed on the 23rd of March 2020. To address the study’s hypotheses: (1) weekly levels of total physical activity significantly reduced (−15%, *p* < 0.001) and (2) daily sedentary behaviour time significantly increased (+33%, *p* < 0.001).

The requirement to remain at home unless for essential reasons during the initial UK lockdown resulted in reduced self-reported day-to-day and recreational physical activity, travel via foot/cycle, and increased sedentary behaviour. The lockdown restrictions and changing individual circumstances likely created barriers relating to the motivation and opportunity to engage in physical activity. Reduced opportunities to travel and the requirement to work from home may have also contributed to the increase in sedentary behaviour. It is apparent that reduced physical activity and increased sedentary behaviour is a negative ‘side-effect’ that occurred during the initial UK lockdown; this is a cause for concern due to the health-related risks associated with these behaviours [8–13]. Cross-sectional research during the COVID-19 pandemic has identified significant associations between reduced physical activity levels and (independently) sedentary behaviour on poorer physical and mental health outcomes [25–27]. Because the first UK COVID-19 lockdown was enforced at a national level these general negative effects are likely to have been experienced by millions of people. Our findings are supported by other related COVID-19 research where data from periods of imposed national lockdowns in the UK and other countries show increased physical inactivity and sedentary time [25–28]. In residents of France and Switzerland, during their respective COVID-19 lockdowns, although vigorous-intensity physical activity decreased and sedentary behaviour increased, there was a concomitant increase in moderate-intensity physical activity participation [25]. This is contrary to the present study where weekly moderate-intensity recreational activities were significantly reduced. Whilst this may potentially reflect differences in restrictions and/or cultural responses to a lockdown, it may also be due to differences in statistical analysis methods, as we employed non-parametric tests due to our data not being normally distributed.

For all sub-group analyses conducted in the present study, no population displayed an increase in overall physical activity (see Table 3) and some displayed non-significant changes, such as individuals living in a town or village, non-athletes, or those that were partnered. Sub-group analysis identified that reductions in total physical activity levels were significantly greater (independently) for those that were single (compared to a relationship), identified as a competitive athlete (compared to non-athlete), and with an average household income < £25,000 (compared to > £25,000). Despite failing to reach statistical significance (*p* = 0.056), there was an interesting trend for the changes in physical activity levels across living locations; effect sizes (ES) for those living in cities, towns, and villages were 0.53, 0.35, and 0.15, respectively, indicating that those living in urbanized places became disproportionally more physically inactive during the lockdown. Sub-group analysis for changes in sedentary time during lockdown also revealed that some populations became significantly more sedentary than their counterparts; namely, those that were single (compared to in a relationship) and those that had an average household income < £25,000 (compared to those with > £25,000). Periods of altered activity become particularly detrimental to long-term health when physical activity participation and sedentary time no longer meet WHO guidelines for optimal health [7]. However, relative to objective measures of physical activity and sedentary time (e.g., accelerometers), participants have been previously shown to overestimate physical activity and underestimate sedentary time when completing the WHO GPAQ [29]. It is, therefore, difficult to establish firm conclusions around the impact of lockdown on adherence to WHO physical activity and sedentary beahviour guidelines.

As the UK government continues to remove public health measures originally put in place to slow the spread of COVID-19, returning to a physically active lifestyle is of paramount importance to much of the population that was negatively impacted by the lockdown restrictions. Official guidelines from the WHO to engage in at least 150–300 min of moderate intensity or 75–150 min of vigorous-intensity physical activity (or some combination of both) per week, plus strengthening activities at least twice a week, should be used as the benchmark for leading a physically active life [7]. Importantly, the guidelines acknowledge that even a few minutes of physical activity can incur positive health benefits [7]. Small modifications to daily habits (e.g., carrying the groceries or using the stairs rather than the lift) should, therefore, be encouraged to help people increase activity levels in a practical and achievable manner [30]. This is particularly important as working from home has now become more normalized and could create barriers in returning to physical activities and reducing the sedentary time that occurred during the lockdown, for example, in people who were previously required to commute but whose jobs can now be partially or completed conducted at home.

 Health practitioners are recommended to consult and employ a risk-stratification approach when overseeing a patient or client’s return to physical activity after COVID-19 infection/illness; readers are directed to Salman et al. [30] for more information on this matter.

### Strengths and limitations

The strengths of this study are that: (1) important cross-sectional data were collected from a large sample of UK adults during the initial COVID-19 lockdown, and (2) individuals with recent changes in health status (e.g., COVID-19 illness) that may have influenced physical activity or sedentary behaviour were excluded from the analyses.

The main limitation of the present study was the retrospective collection of pre-lockdown data. To reduce the risk of recall bias, simple questions and fast-tracking research design, ethical approval, survey dissemination, and survey closure were implemented. However, given the fast-evolving nature of the pandemic in its early stages, retrospective data collection was the only available method available to retrieve this data. Further, it was logistically and financially difficult to collect direct measures of physical activity (e.g., accelerometers), and therefore indirect self-reported measures were collected through an online survey. Whilst this improved the available sample size, the present study’s findings should be correctly interpreted as estimates. As the survey was disseminated via snowball sampling, self-selection bias may have led to an overrepresentation of a population that had more free time to complete an online survey. Therefore, the findings may underrepresent busy populations such as keyworkers, overtime workers, or those with heightend levels of responsibilities (childcare, home schooling, caring, etc.).

## Conclusion

During the initial month of the first UK lockdown (enforced on the 23rd of March 2020) in response to the COVID-19 pandemic, self-reported levels of physical activity significantly decreased (−15%, p < 0.001) and sedentary behaviour significantly increased (+33%, p < 0.001) in UK adults compared to before. Now that the UK is transitioning to a phase of normal living, it is important that individuals are encouraged and supported to gradually return to/increase levels of physical activity, using 2020 WHO physical activity and sedentary behaviour guidelines for goal setting. Modifications to daily habits (e.g., carrying the shopping, using the stairs instead of a lift, or a standing desk) should be acknowledged as effective and practical ways to increase physical activity levels and/or reduce sedentary behaviours for the promotion of one's health. A risk-stratification approach is recommended when returning individuals that have been infected by COVID-19 to physical activity to minimize health risks.

## Data Availability

Data supporting the present study are available in the online public repository Zenodo at: https://doi.org/10.5281/zenodo.4501530
