# Correction to: COVID-19: self-reported reductions in physical activity and increases in sedentary behaviour during the first national lockdown in the United Kingdom

**DOI:** 10.1007/s11332-023-01057-9

**Published:** 2023-04-08

**Authors:** Patrick Swain, Emily James, Jonathan M. Laws, Clare Strongman, Stuart Haw, Gill Barry, Henry C. Chung, Dan Gordon

**Affiliations:** 1 Faculty of Health and Life Sciences, Northumbria University, Newcastle-upon-Tyne, UK; 2 Cambridge Centre for Sport and Exercise Sciences, Anglia Ruskin University, Cambridge, UK

**Correction to: Sport Sciences for Health** 10.1007/s11332-022-01012-0

The reference no. 4 has been incorrectly published in the original publicaiton. The complete correct reference [4] is given below.

4. Chen P et al (2020) **Coronavirus disease (COVID-19)**: the need to maintain regular physical activity while taking precautions. J Sport Health Sci 9(2):103.

